# Pneumatosis cystoides intestinalis presenting as pneumoperitoneum in a patient with chronic obstructive pulmonary disease: a case report

**DOI:** 10.1186/s13256-017-1198-2

**Published:** 2017-02-28

**Authors:** Atsuyoshi Iida, Hiromichi Naito, Kohei Tsukahara, Tetsuya Yumoto, Nobuyuki Nosaka, Shinnichi Kawana, Keiji Sato, Nobuhiro Takeuchi, Jyunichi Soneda, Atsunori Nakao

**Affiliations:** 1 Department of Emergency and Critical Care Medicine, Okayama University Graduate School of, Medicine, Dentistry and Pharmaceutical Sciences, 2-5-1 Shikata-cho, Kita-ku, Okayama-shi, Okayama 700-8558 Japan; 2 Department of Emergency Medicine, Kobe Tokusyukai Hospital, Kobe, Japan

**Keywords:** Computed tomography, Intramural gas, Intraperitoneal free air, Benign disease

## Abstract

**Background:**

Pneumatosis cystoides intestinalis, marked by numerous gas-filled cysts in the intestinal wall and submucosa or intestinal submucosa, is a very uncommon condition.

**Case presentation:**

A 79-year-old Asian man presented to our emergency department after 2 days of lower abdominal pain with nausea and constipation. His past medical history included chronic obstructive pulmonary disease and he had been treated with home oxygen therapy. The patient was hemodynamically stable and had mild generalized abdominal pain and a soft, distended abdomen without signs of peritonism. A computed tomography scan showed diffuse intraluminal gas and intraperitoneal free gas. Based on the images, a clinical diagnosis of pneumatosis cystoides intestinalis with pneumoperitoneum was made. Considering the patient’s physical examination, the peritoneal free air was drained by aspiration and he was observed for 12 h, but remained well. Abdominal symptoms and pneumoperitineum resolved after drainage of the peritoneal air by aspiration. The suspected etiopathogenic mechanism of pneumatosis cystoides intestinalis in the presented patient may have been alveolar air leakage secondary to high airway pressure due to chronic obstructive pulmonary disease; air leakage from an alveolar rupture may have traveled to the retroperitoneum through the mediastinal vessels and entered the mesentery of the bowel.

**Conclusion:**

Emergency physicians should be aware of the potential development of pneumatosis cystoides intestinalis in chronic obstructive pulmonary disease patients.

## Background

Pneumatosis cystoides intestinalis (PCI) is an uncommon condition, characterized by the presence of multiple gas-filled cysts within the submucosa or subserosa of the gastrointestinal tract. This sign is usually found on radiographic imaging and it most commonly secondary to acute intestinal ischemia. PCI has diverse clinical outcomes ranging from benign, which does not require treatment, to a septic condition that can lead to death.

We report a case of PCI presenting as pneumoperitoneum, occurring concomitantly with chronic obstructive pulmonary disease (COPD) in combination with prolonged intestinal distention.

Although detection of free abdominal air in most cases requires immediate surgical intervention, our case was successfully treated without emergency laparotomy. Our case report will help the readers to increase their understanding of PCI. Conservative care should be considered first if the patient is stable, while emergency laparotomy should be reserved for life-threatening abdominal pathology.

## Case presentation

A 79-year-old Asian man presented to our emergency department with a 2-day history of lower abdominal pain with nausea and constipation. His past medical history included chronic obstructive pulmonary disease (COPD) and had been treated with home oxygen therapy. The patient was hemodynamically stable and had mild generalized abdominal pain but with a soft distended abdomen. There were no signs of peritonism. Physical examination on admission revealed the following findings: blood pressure, 104/60 mmHg; heart rate, 68 beats/min; body temperature, 37.1 °C.

**Fig. 1:**
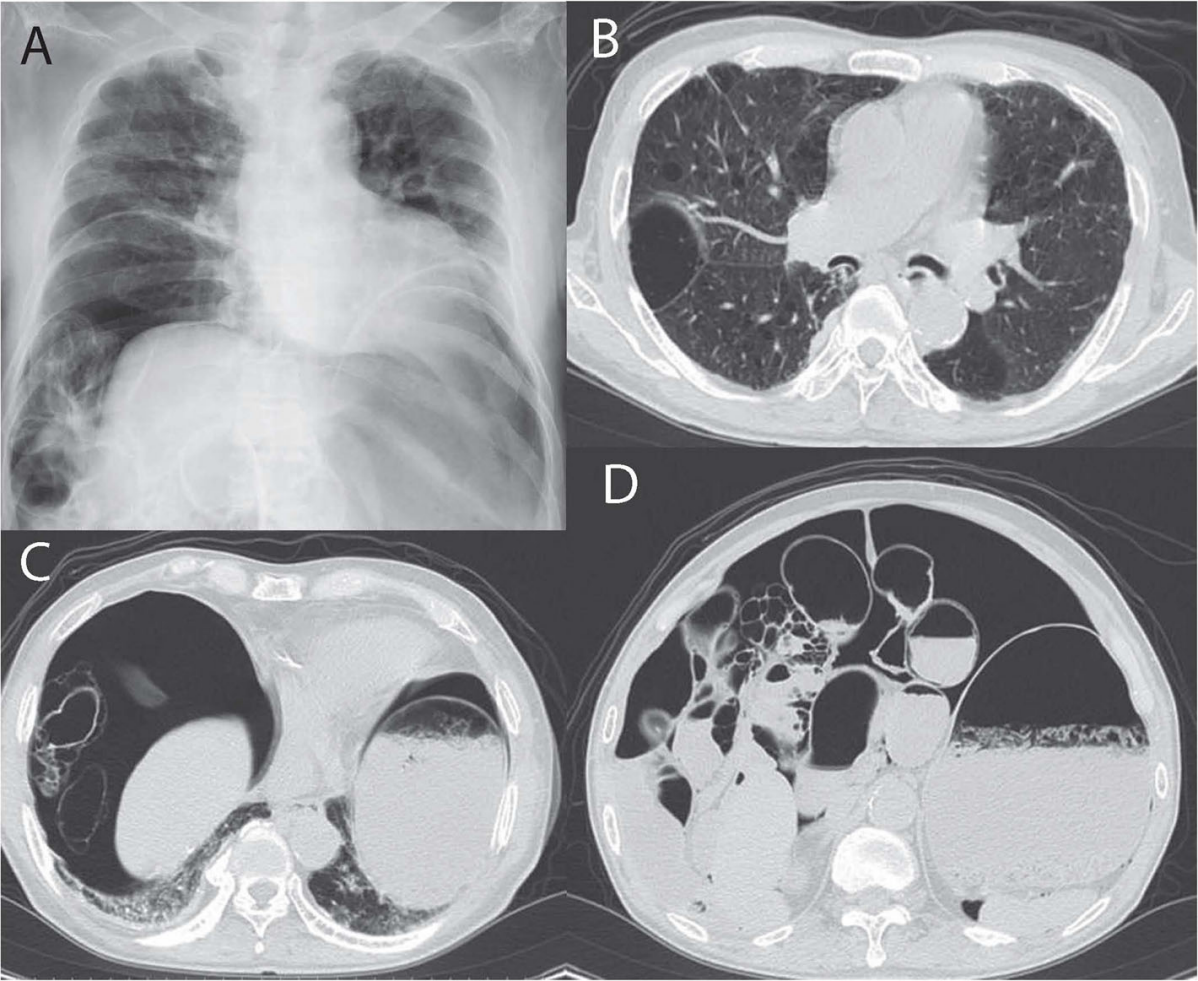
**a** Plain chest radiograph taken on admission indicated pneumoperitoneum. **b** Chest computed tomography (CT) revealed pulmonary emphysema with multiple cystic changes. **c**, **d** Abdominal and pelvic CT demonstrated pneumatosis cystoides intestinalis in the ileum and distal ascending colon associated with pneumoperitoneum

Blood tests showed a white blood cell count of 12,840/mm^3^ and a C-reactive protein level of 1.55 mg/dL. A computed tomography (CT) scan showed diffuse intraluminal gas and intraperitoneal free gas, indicating PCI in the ileum and the proximal ascending colon to the distal transverse colon. Chest CT revealed pulmonary emphysema with multiple cystic changes. Based on the images, a clinical diagnosis of PCI with pneumoperitoneum was made (Fig. 1). Considering the patient’s physical examination, the peritoneal free air was drained by aspiration and he was observed for 12 h, but remained well. Abdominal symptoms and pneumoperitineum resolved after aspiration of the peritoneal air and the patient was discharged after 13 days.

## Discussion

PCI, known also as intramural gas, refers to gas within the bowel wall. Alternative names include pseudopneumatosis, intestinal emphysema, and bullous emphysema of the intestine. The overall incidence of PCI is 0.3% as revealed by the increased use of CT. PCI can be divided into two general types: primary and secondary. The primary or idiopathic type, which is not associated with other coexisting diseases, accounts for about 15% of cases. The secondary type comprises the remaining 85% of cases [1, 2].

The etiology of PCI is not entirely clear and a single mechanism cannot account for all cases.

Several theories, including mechanical, pulmonary, bacterial, and biochemical, have evolved to help explain the etiology. The mechanical theory is that gas migrates from the gastrointestinal lumen into the submucosal or subserosal layer of the intestinal wall. Increased intraluminal pressure forces gas within the bowel lumen to breach the mucosal or serosal layers, accumulating to form cysts. Vomiting or intestinal obstruction can increase intraluminal pressure, causing mechanical injury to the intestinal wall and a break in the mucosa, which allows gas migration.

The pulmonary hypothesis attributes increased intraluminal pressure to the respiratory system [3, 4]. The theory is that air leakage from an alveolar rupture travels to the retroperitoneum through the mediastinum and retroperitoneum and locates within the bowel mesentery. The third hypothesis, the bacterial theory, proposes that bacteria form gas in the gastrointestinal wall, the gas then migrates into the intestinal wall contributing to the development of PCI [2].

The submucosal localization of fermenting bacteria, such as Clostridia and *Escherichia coli*, leads to the production of gas that subsequently accumulates in the submucosa. The biochemical theory is that the increased production of hydrogen gas during carbohydrate metabolism raises the intraluminal pressure which forces the gas into the weakened bowel wall.

Likewise, the use of alpha-glucosidase inhibitors to treat type 2 diabetes mellitus has been found to be associated with PCI [5].

Since our patient had a history of longstanding COPD, we assumed that constant coughing had caused alveolar rupture. In such cases, alveolar gas dissects along the aorta within the mediastinum, through the diaphragm and into the mesenteric blood vessels, breaching the bowel wall and then becoming trapped in the bowel wall to form cysts. Our medical literature search yielded several previous case reports of PCI presumably caused by the pulmonary theory mechanism [6, 7]. These cases presented similar clinical characteristics and did not require laparotomy as seen in the present case.

Most patients with PCI have distention, abdominal pain, nausea, and vomiting. The most frequent symptoms are abdominal pain, diarrhea, abdominal distension, constipation, and tenesmus, as well as loss of appetite, excessive flatulence, and weight loss. These nonspecific symptoms can easily lead to the wrong diagnosis of irritable bowel syndrome. Ordinarily, in adults, PCI is a benign disease that spontaneously regresses, although diagnosis of coexisting disease(s) is usually evident.

Appropriate therapy is associated with the underlying cause of PCI. For at least 50% of patients, no treatment is necessary. PCI may disappear spontaneously, persist for many years, or reoccur after treatment. A few days of an elementary diet, antibiotic therapy, or a high concentration of oxygen or hyperbaric oxygen can assist the recovery of patients with serious gastrointestinal symptoms, although low-concentration oxygen is also clinically beneficial [8, 9]. Wayne *et al.* postulated a clinical algorithm to identify subgroups to direct surgical intervention for acute ischemic insults and to prevent nontherapeutic laparotomies being undertaken for benign PCI [8]. However, we do not impose our therapeutic strategy of aspiration of the peritoneal air as the standard; removal of the peritoneal air may not be necessary.

## Conclusion

Emergency physicians should be aware of the potential development of PCI in COPD patients. Our case stresses that conservative treatment of pneumoperitoneum for secondary PCI can yield satisfactory outcomes, while emergency laparotomy should be reserved for life-threatening abdominal pathology.

## Abbreviations

COPD: Chronic obstructive pulmonary disease
CT: Computed tomography
PCI: Pneumatosis cystoides intestinalis
